# Anti-CAR-engineered T cells for epitope-based elimination of autologous CAR T cells

**DOI:** 10.1007/s00262-019-02376-y

**Published:** 2019-08-14

**Authors:** Stefanie Koristka, Pauline Ziller-Walter, Ralf Bergmann, Claudia Arndt, Anja Feldmann, Alexandra Kegler, Marc Cartellieri, Armin Ehninger, Gerhard Ehninger, Martin Bornhäuser, Michael P. Bachmann

**Affiliations:** 1 Department of Radioimmunology, Institute of Radiopharmaceutical Cancer Research, Helmholtz-Zentrum Dresden-Rossendorf (HZDR), Bautzner Landstraße 400, 01328 Dresden, Germany; 2 Tumor Immunology, University Cancer Center (UCC), ‘Carl Gustav Carus’ Technische Universität Dresden, Fetscherstraße 74, 01307 Dresden, Germany; 3 Cellex Patient Treatment GmbH, Tatzberg 47, 01307 Dresden, Germany; 4 GEMoaB Monoclonals GmbH, Tatzberg 47, 01307 Dresden, Germany; 5 Medical Clinic and Policlinic I, University Hospital ‘Carl Gustav Carus’ Technische Universität Dresden, Fetscherstraße 74, 01307 Dresden, Germany; 6 German Cancer Consortium (DKTK), partner site Dresden, and German Cancer Research Center (DKFZ), Heidelberg, Germany; 7 National Center for Tumor Diseases (NCT), ‘Carl Gustav Carus’ Technische Universität Dresden, Dresden, Germany

**Keywords:** Chimeric antigen receptor, Immunotherapy, Toxicity management, Elimination tag

## Abstract

Although CAR T-cell therapy has demonstrated tremendous clinical efficacy especially in hematological malignancies, severe treatment-associated toxicities still compromise the widespread application of this innovative technology. Therefore, developing novel approaches to abrogate CAR T-cell-mediated side effects is of great relevance. Several promising strategies pursue the selective antibody-based depletion of adoptively transferred T cells via elimination markers. However, given the limited half-life and tissue penetration, dependence on the patients’ immune system and on-target/off-side effects of proposed monoclonal antibodies, we sought to exploit αCAR-engineered T cells to efficiently eliminate CAR T cells. For comprehensive and specific recognition, a small peptide epitope (E-tag) was incorporated into the extracellular spacer region of CAR constructs. We provide first proof-of-concept for targeting this epitope by αE-tag CAR T cells, allowing an effective killing of autologous E-tagged CAR T cells both in vitro and in vivo whilst sparing cells lacking the E-tag. In addition to CAR T-cell cytotoxicity, the αE-tag-specific T cells can be empowered with cancer-fighting ability in case of relapse, hence, have versatile utility. Our proposed methodology can most probably be implemented in CAR T-cell therapies regardless of the targeted tumor antigen aiding in improving overall safety and survival control of highly potent gene-modified cells.

**Electronic supplementary material:**

The online version of this article (10.1007/s00262-019-02376-y) contains supplementary material, which is available to authorized users.

## Introduction

Within the last decades, the tremendous progress in genetic engineering paved the way for generating CAR T cells. CARs typically comprise an extracellular antigen recognition moiety fused via a flexible hinge and transmembrane region to an intracellular signaling unit, thereby combining the virtues of Abs (high antigen-binding specificity) and immune cells (potent anti-tumor effector mechanisms) within one single fusion molecule [1]. Especially for the treatment of relapsed/refractory B-cell-derived malignancies, CAR T-cell therapy targeting the CD19 antigen has achieved remarkable clinical results [2–6].

However, severe treatment-associated toxicities still restrain the widespread application of this promising technology. The most frequent side effects following CAR T-cell administration include cytokine release syndrome [4–7], neurotoxicity [6, 7], on-target/off-tumor responses [4, 8–10], and anaphylaxis [11], all of which may lead to life-threatening or even fatal implications. Given the enormously long-term persistence and proliferative capacity of genetically engineered T cells [12], control and reversal of toxicity have emerged as important aspects of CAR T-cell therapy.

Various approaches have been pursued to mitigate side effects ranging from global, unspecific immunosuppression to selective ablation of CAR-engineered T cells. The latter strategy is currently under massive investigation and is based on the transgenic introduction of either suicide genes or elimination marker genes. Two well-studied suicide genes are herpes simplex virus thymidine kinase [13] and inducible caspase-9 [14–16] which can be triggered by the administration of small molecules to effectively induce CAR T-cell death. Alternatively, forced expression of a targetable cell-surface antigen physiologically not present on T lymphocytes has been evaluated. Proposed elimination markers are truncated EGFR [17, 18] and CD20 [19, 20] which are recognized by the mAbs cetuximab and rituximab, respectively. Infusion of these therapeutic molecules subsequently results in specific CAR T-cell depletion via antibody-dependent cellular cytotoxicity (ADCC) and complement-dependent cytotoxicity (CDC).

Although all of the above-mentioned methodologies allow the effective elimination of adoptively transferred T cells in case of severe toxicities, each approach exhibits distinct limitations potentially restricting a broad clinical utility. These include immunogenicity [21], large size of the proposed depletion marker (over 130 aa), dependence on the patients’ immune system (ADCC, CDC) and occurrence of on-target adverse events due to mAb recognition of healthy tissue [22]. Moreover, all techniques are based on the insertion of an additional gene that is co-expressed with the CAR of interest, bearing the risk of CAR T-cell escape in case the safety switch is not uniformly and/or stably expressed. Therefore, we intended to use a short peptide epitope (E-tag) directly incorporated into the CAR architecture as a targetable moiety for selective and stringent CAR T-cell elimination [23, 24]. Another substantial drawback of currently available safeguard strategies is their reliance on pharmacological drugs whose therapeutic effect inevitably declines due to their short half-life. For that reason, we sought to use T lymphocytes as living drugs that are equipped with a CAR construct directed against a targetable portion of the therapeutic CAR. In this report, we demonstrate the utility of the E-tag as a selection marker for an efficient T-cell-mediated elimination of autologous CAR T cells.

## Materials and methods

### Cell lines and culture

All cell lines were kept at 37 °C in a humidified atmosphere of 5% CO_2_. PC3 cells were stably transduced with the open reading frame (orf) encoding the prostate stem cell antigen (PSCA) as previously reported [25]. The murine fibroblast cell line 3T3 and the human embryonic kidney cell line HEK293T were cultured in DMEM (ThermoFisher Scientific, Schwerte, Germany) supplemented with 10% FCS, 100 µg/ml penicillin/streptomycin and 1% non-essential aa. PC3-PSCA and Chinese hamster ovary (CHO) cells were maintained in RPMI 1640 media supplemented with 10% FCS, 100 µg/ml penicillin/streptomycin, 1% non-essential aa, 1 mM sodium pyruvate and 2 mM *N*-acetyl-l-alanyl-l-glutamine (all purchased from Biochrom, Berlin, Germany).

### Generation of CAR vectors

Cloning and structural composition of conventional CARs as well as the universal chimeric antigen receptor (UniCAR) 28/ζ construct have been described previously in detail [23, 24, 26]. Briefly, they contain an extracellular binding moiety derived from the αPSCA MB1 mAb [25], the αCD19 HD37 mAb [27] or the αLa 5B9 mAb [28] followed by hinge and transmembrane domain of human CD28 and cytoplasmic activation domains of CD28 and CD3ζ. As a targetable moiety, the peptide epitope E7B6 (E-tag) is incorporated in the extracellular spacer region. The Stop variant of the UniCAR lacks intracellular sequences downstream of the CD28 transmembrane domain.

To generate a UniCAR lacking the E-tag, the DNA fragment coding for the extracellular part of the CAR 28/ζ was cut out of the respective vector with *Sfi*I and *Hpa*I. A DNA sequence encoding the scFv 5B9 and hinge region without the E7B6 tag was synthesized by Eurofins Genomics (Ebersberg, Germany) and cloned via *Sfi*I and *Hpa*I into the expression vector p6NST60-CAR 28/ζ, generating the ΔCAR 28/ζ construct.

The novel αE-tag CAR 28/ζ was obtained as follows: first, a fragment encoding the antigen-binding domain was generated by cutting the sequence of the humanized scFv La (7B6) *V*_L_–*V*_H_ out of a pSecTag2B vector with *Nhe*I and *Apa*I. The orf of a fragment containing the hinge, transmembrane and signaling domain of human CD28 as well as human CD3ζ signaling chain was cut out of a cloning vector available in our lab using the restriction enzymes *Mss*I and *Apa*I. Finally, the expression vector p6NST60-MCS was digested with *Xba*I/*Ksp*AI and ligated with the aforementioned two fragments resulting in the vector p6NST60-hu αE-tag CAR 28/ζ.

The Orf of all CAR constructs was C-terminally fused to the DNA sequence coding for enhanced green fluorescent protein (EGFP). To allow for a co-translation of CAR and EGFP from one mRNA, both reading frames are separated by a 2A ‘cleavage’ site derived from Thosea asigna virus that induces a ribosomal ‘skip’ from one codon to the next without the formation of a peptide bond [29].

### Production and purification of αPSCA targeting modules (TMs)

Design, cloning, and purification of αPSCA-E5B9 TM from 3T3 cell culture supernatants was previously described in detail [25, 30]. For redirection of αE-tag CAR T cells, murine or humanized αPSCA scFvs (clone MB1) were C-terminally modified to contain the E7B6 epitope (E-tag). The respective orfs were cloned into the p6NST50 vector which was used to generate a stable CHO cell line for permanent TM production. Purification from cell culture supernatants and analysis of protein concentration were conducted as reported elsewhere [25, 30, 31].

### Isolation, transduction, and expansion of human primary T cells

Buffy coats were supplied by German Red Cross (Dresden, Germany). Human CD3^+^, CD4^+^ or CD8^+^ T cells were isolated and cultured as described previously [25, 28, 30, 31].

Production of lentiviral particles using HEK293T cells and T-cell transduction was conducted as reported elsewhere [23, 26, 32]. In brief, T cells were stimulated using αCD3/CD28 DynaBeads^®^ (Invitrogen, ThermoFisher Scientific) at a 1:4 bead to cell ratio. For T-cell transduction concentrated virus supernatant was added five times within the first 3 days of expansion. After successful genetic modification verified by EGFP expression, cells were sorted on a FACSAria III (BD Biosciences Pharmingen, Heidelberg, Germany). One day prior to experiments, T cells were rested in complete RPMI 1640 lacking any recombinant cytokines.

### Flow cytometric analysis

Flow cytometry was carried out on a MACSQuant^®^ Analyzer (Miltenyi Biotec, Bergisch Gladbach, Germany) and acquired data were analyzed using MACSQuantify^®^ Software (Miltenyi Biotec) or FlowJo 10.1 Software (TreeStar Inc., Ashland, OR USA). Fluorescently labeled mAbs directed against human CD3 (clone BW264/56), CD4 (clone REA623), CD8 (clone REA734), CD69 (clone FN50), granzyme A (GzmA, clone REA162) and granzyme B (GzmB, clone REA226) as well as respective REA isotype controls (clone REA293) were purchased from Miltenyi Biotec. mAbs against human CD107a (clone H4A3) and IgG1 (isotype control, clone MOPC-21) were obtained from BD Biosciences Pharmingen. For the detection of E-tagged CAR constructs on genetically modified T cells, the αE-tag mAb (clone 7B6) and secondary goat anti-mouse IgG F(ab′)2-PE Ab (Beckmann Coulter, Krefeld, Germany) were used [23].

After staining of extracellular markers and live vs. dead cell discrimination applying a Zombie Red™ Fixable Viability Kit (BioLegend, London, UK), T cells were processed using an Inside Stain Kit (Miltenyi Biotec) according to the manufacturer’s protocol and subsequently labeled for intracellular markers.

### Cytotoxicity assays

The elimination of CAR T cells by αE-tag CAR T cells was assessed using a previously established flow cytometry-based assay [33, 34]. To distinguish effector from target cells, the latter were labeled with 10 µM cell proliferation dye eFluor™450 (eBioscience, ThermoFisher Scientific) according to the manufacturer’s instructions. The next day, effector cells (αE-tag CAR T cells) were incubated with eFluor™450-stained CAR 28/ζ, ΔCAR 28/ζ or CAR Stop T cells at indicated effector to target cell (*E*:*T*) ratios. After indicated time points, cocultures were carefully resuspended and an aliquot of 25 µl diluted 1:4 with 1 µg/ml propidium iodide (Sigma-Aldrich, Munich, Germany) was measured at a MACSQuant^®^ Analyzer. After exclusion of doublets and dead cells, cells/ml were assessed for both effector and target T cells separately. Finally, cells of one triplet were pooled, stained for intracellular expression of GzmA and GzmB, and analyzed by flow cytometry.

The antitumor activity of αE-tag CAR T cells was determined in standard chromium-51 release assays as previously reported [25].

### T-cell activation and degranulation assay

A total of 5x10^5^ eFluor™450-labeled CAR 28/ζ, ΔCAR 28/ζ or CAR Stop T cells was cultured together with equal numbers of αE-tag CAR T cells. Additionally, 5 µl of αCD107a mAb or IgG1 isotype control were pipetted to each well. After 1 h of incubation at 37 °C, 1 µl of 2 mM monensin (Sigma-Aldrich) was added. After 20 h, cells were harvested, stained with αCD69 and αCD3 mAb and measured using a MACSQuant^®^ Analyzer. Living cells were distinguished from dead cells by being propidium iodide negative.

### Cytokine release assay

For analysis of TNF, IFN-γ, GM-CSF and IL-2 concentrations in cell-free culture supernatants (stored at − 80 °C) enzyme-linked immunosorbent assay (ELISA) was conducted using OptEIA™ Human ELISA Kits (BD Biosciences Pharmingen) according to the manufacturer’s protocol.

### Optical imaging of tumor xenograft mice

Five-week-old male NMRI-Foxn1^nu^/Foxn1^nu^ mice were s.c. injected into their right flank with 5 × 10^5^ firefly luciferase (Luc)-expressing PC3-PSCA cells (*n* = 5 per group). Control animals received tumor cells only or tumor cells mixed with either 5 × 10^5^ CAR 28/ζ T cells or 15 × 10^5^ αE-tag CAR T cells. For induction of anti-tumor activity, 10 µg of E5B9- or E7B6-tagged αPSCA TM was administered in addition to tumor cells and CAR-engrafted T cells. To impair the anti-tumor response, one group of mice was transplanted with PC3-PSCA-Luc cells, 10 µg αPSCA-E5B9 TM, 5 × 10^5^ E-tagged CAR target T cells, and 15 × 10^5^ αE-tag CAR effector T cells. Animals were anesthetized 6 h, 1 day and 2 days post-cell mixture injection and bioluminescence imaging was conducted as described elsewhere [26, 32]. Optical images were created with Bruker Multispectral software (Bruker, Germany).

### Statistical analysis

Statistical significance was evaluated with GraphPad Prism 7 software (GraphPad Software Inc., La Jolla, CA, USA). Statistical tests were used as indicated in figure legends. *p* values of less than 0.05 were considered significant.

## Results

### Generation of an αCAR CAR construct for the depletion of CAR-expressing T cells

**Fig. 1:**
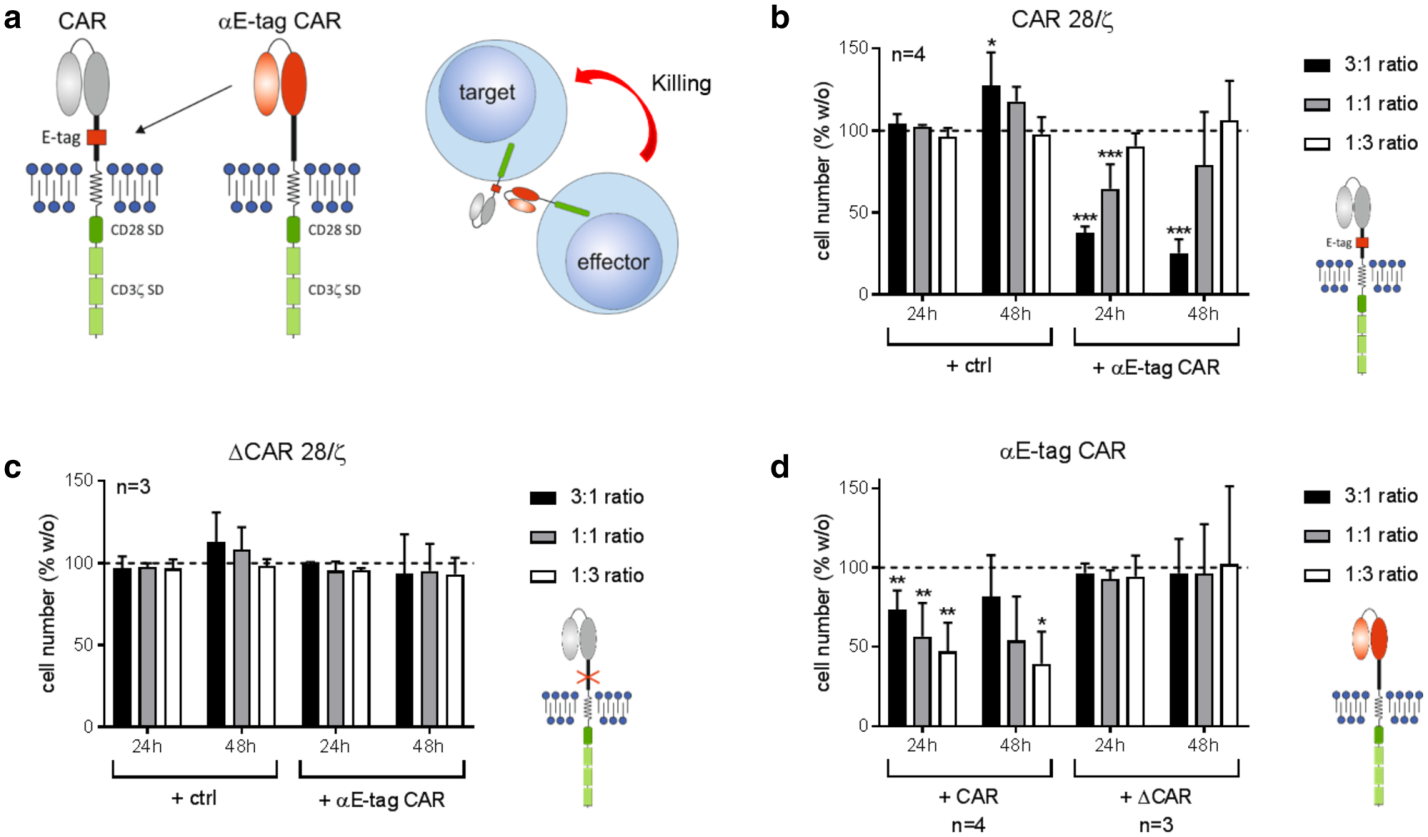
Elimination of CAR 28/ζ T cells by αE-tag CAR effector T cells. **a** Schematic representation of an αE-tag CAR and its mode of action. **b**–**d** UniCAR-modified T cells either containing (CAR 28/ζ) or lacking (ΔCAR 28/ζ) the extracellular E-tag were incubated with αE-tag CAR effector or mock-transduced (ctrl) T cells at indicated *E*:*T* ratios. Diagrams show cell number of **b** eFluor™450^+^ CAR 28/ζ T cells, **c** eFluor™450^+^ ΔCAR 28/ζ T cells, or **d** unstained αE-tag CAR effector cells. Absolute cell numbers alone were set to 100% and relative cell number in the presence of effector/target cells was calculated. Statistical significance was determined by one-way ANOVA with Bonferroni multiple comparison test (*p < 0.05, **p < 0.01, ***p < 0.001 with respect to T cells cultured alone)

For targeting of CAR-modified T cells, we compared and tested different short peptide tags that can be incorporated into the extracellular part of both our conventional CARs as well as our UniCARs (data not shown). Thereby, we identified a short epitope derived from the nuclear protein La/SS-B (E-tag) as the most suitable tag for our approach. It could be integrated without impairing the in vitro or in vivo functionality of CAR T cells as previously published [23, 24, 26, 27, 32, 35–38]. The 18-aa-long sequence E7B6 (EKEALKKIIEDQQESLNKW) is specifically bound by the αLa 7B6 mAb [39]. Consequently, we cloned a CAR with a 7B6-derived scFv as the antigen-binding moiety and termed the resulting αCAR construct αE-tag CAR (Fig. 1a). The αE-tag CAR construct recognizes the peptide tag E7B6 located in the hinge region of CARs. Upon antigen recognition, αE-tag CAR effector cells are cross-linked to target cells, which should result in the elimination of the latter. For signal transduction, the newly generated CAR contains the activating cytoplasmic domains of CD3 and CD28. Isolated CD3^+^ T cells from healthy donors could be successfully modified to express the novel CAR with CD4^+^ and CD8^+^ subpopulations yielding comparable transduction rates (supplementary Fig. 1a).

### Specific elimination of CAR T cells using αE-tag CAR-engineered T cells

To test functionality of the novel αE-tag CAR construct, flow cytometry-based cytotoxicity assays were performed. As target cells, T lymphocytes were used that express a UniCAR containing the extracellular E-tag and an intracellular 28/ζ signaling domain. As shown in Fig. 1b, culturing these cells together with mock-transduced control cells did not influence viability. In contrast, adding αE-tag CAR effector T cells resulted in significant elimination of target T cells. Expectedly, T-cell killing was dependent on the *E*:*T* ratio. To verify that the observed cytotoxic effect is due to specific recognition and binding of the incorporated peptide epitope, experiments with E-tag-deleted CAR T cells (termed ΔCAR 28/ζ) were conducted (Fig. 1c). As anticipated, ΔCAR 28/ζ-engineered lymphocytes were not targeted by αE-tag CAR effector T cells. In addition, we monitored the number of living effector T cells after 24 h and 48 h of coculture. To our surprise, T cells redirected against the E-tag were significantly reduced in cell number whilst viability was maintained in the presence of ΔCAR 28/ζ-armed target cells (Fig. 1d). Interestingly, this effect inversely correlated with the chosen *E*:*T* ratio. To confirm these results, we, furthermore, tested whether E-tagged CAR constructs with different antigen specificity can be targeted as well. In accordance with the data obtained for UniCAR-armed target cells, T cells expressing an αCD19 or an αPSCA conventional CAR (containing the extracellular E-tag) were specifically eliminated upon coculture with αE-tag CAR effector T cells already after 24 h (supplementary Fig. 1b). Again, survival of αCAR-redirected effector cells was affected by coculture with target cells in an *E*:*T*-dependent manner.

**Fig. 2:**
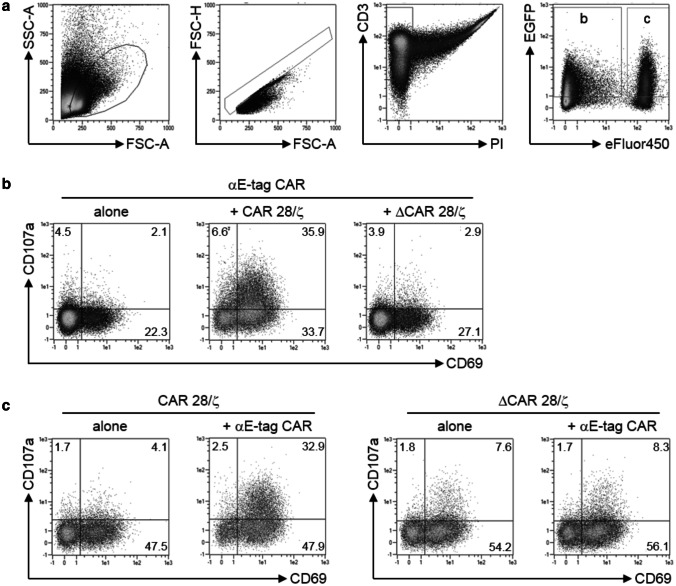
Analysis of activation and degranulation of effector and target T cells. Target CAR T cells harboring (CAR 28/ζ) or lacking (ΔCAR 28/ζ) the E-tag were cultured with αE-tag CAR effector T cells at an *E*:*T* ratio of 1:1. One day later, cells were analyzed for expression of CD69 as well as CD107a. **a** Gating hierarchy starting with a lymphocyte scatter gate followed by exclusion of doublets, then gating on living T cells and finally on EGFP^+^eFluor450^−^ effector cells and EGFP^+^eFluor450^+^ target cells. **b**, **c** Density plots show percentage of CD69^+^ and/or CD107a^+^**b** effector and **c** target cells for one out of three representative donors

In a next step, we aimed to clarify whether the diminished effector T-cell number was merely a result of T-cell activation/exhaustion or reflects apoptosis actively induced by cross-linked target cells. For that purpose, expression of activation-induced CD69 as well as CD107a was evaluated (Fig. 2). The latter not only represents a marker for degranulation but was also recently described as a marker protein for fratricide of T cells [40]. In accordance with cytotoxicity data, αE-tag CAR effector T cells selectively upregulated both CD69 and CD107a in the presence of E-tag-labeled CAR 28/ζ target cells but not upon coculture with ΔCAR 28/ζ-equipped lymphocytes (Fig. 2b). Regarding CD69 and CD107a on the target cell side, T cells lacking the peptide epitope did not increase expression of neither of the analyzed surface molecules (Fig. 2c). However, target cells harboring the E-tag were stimulated and degranulated upon coincubation with αE-tag CAR effector T cells. These results indicate that cross-linkage of effector and target T cells triggers activation of both cell types and subsequent reciprocal T-cell elimination.

### Reciprocal T-cell killing is dependent on the intracellular signaling domain

**Fig. 3:**
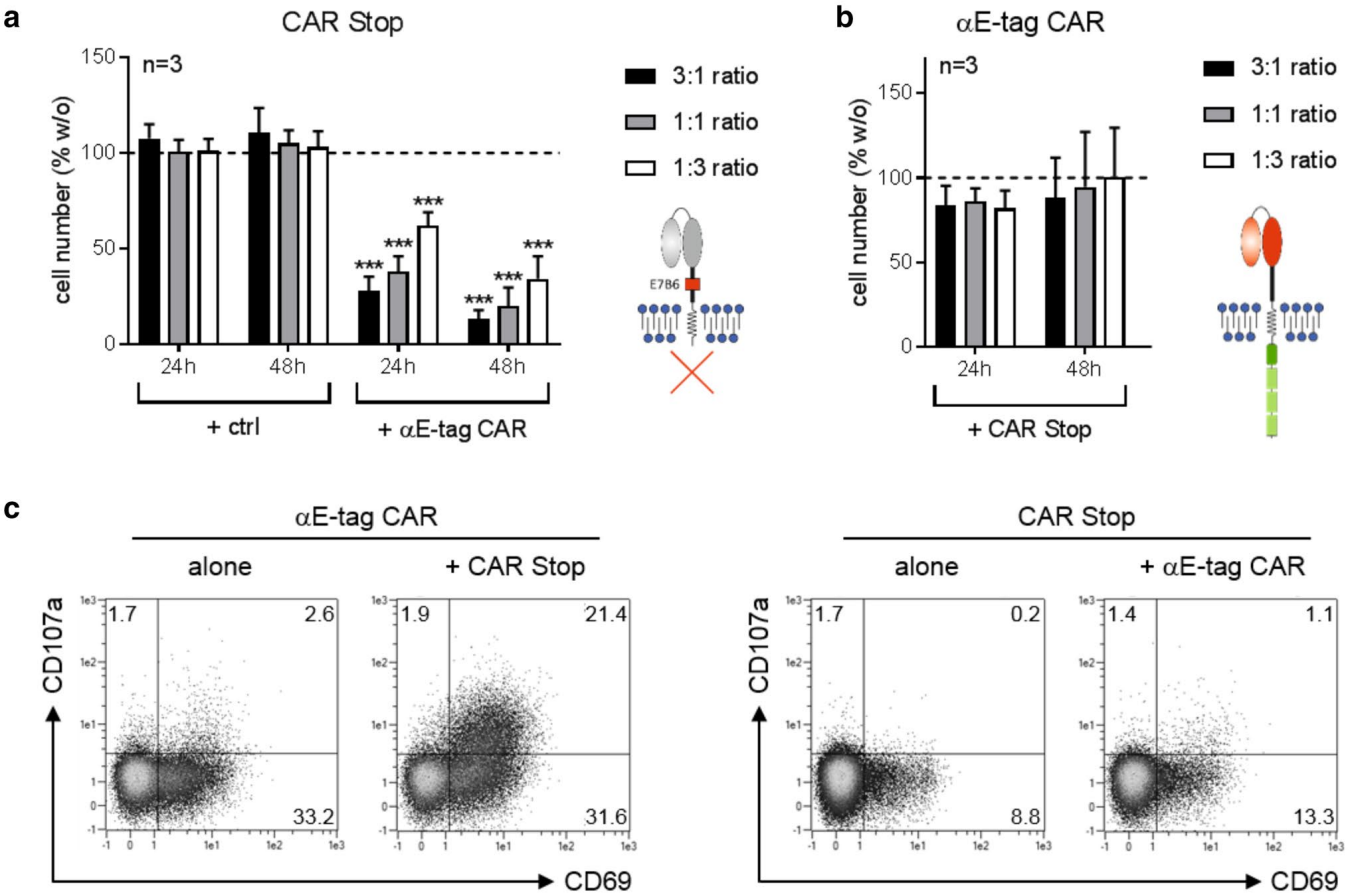
Elimination of CAR Stop T cells by αE-tag CAR T cells. T cells transduced with an E-tagged CAR construct lacking an intracellular signaling domain (CAR Stop) were incubated with αE-tag CAR effector or mock-transduced (ctrl) T cells at indicated ratios. Cell number of **a** eFluor™450-stained target cells or **b** unstained effector cells was assessed after 24 h and 48 h. Number of T cells cultured alone was equalized to 100% and percentage of cells in the presence of effector/target cells calculated (***p < 0.001 with respect to T cells cultured alone; one-way ANOVA with Bonferroni multiple comparison test). **c** Percentage of CD69^+^ and/or CD107a^+^ EGFP^+^ αE-tag CAR effector or CAR Stop target T cells after 24 h of coculture from one out of three representative T-cell donors

The data obtained so far clearly demonstrate functionality of the novel αE-tag CAR construct. However, binding to E-tag-comprising CAR 28/ζ target cells led to fratricide especially at low *E*:*T* ratios. This could either be due to cross-linkage of both cell types and formation of an immune synapse-like structure per se or due to triggering of the intracellular 28/ζ signaling domain of CAR-equipped target cells. To shed light on this issue, we performed cytotoxicity assays with target T cells that express an E-tag-containing CAR construct without an intracellular signaling unit (CAR Stop) (Fig. 3). As depicted in Fig. 3a, these cells were efficiently depleted by αE-tag CAR effector T cells at all *E*:*T* ratios tested, thereby verifying the utility of the E-tag for specific targeting of genetically modified cells. As effector cell numbers remained constant, no reciprocal killing occurred (Fig. 3b). In line with this observation, only redirected effector T cells but not E-tag-labeled target cells were activated and degranulated upon cross-linkage (Fig. 3c). Hence, these data clearly demonstrate that reciprocal elimination of CAR-armed T cells occurs in dependence on an intracellular signaling domain.

To investigate which cell type prevails in coculture, long-term cytotoxicity assays were conducted (supplementary Fig. 2a and 2b). The outcome was both dependent on the targeted CAR construct as well as the *E*:*T* ratio. Cells modified to express an E-tag-containing αCD19 or αPSCA conventional CAR were efficiently eradicated long term at *E*:*T* ratios of 3:1 and 1:1. Compared to this, UniCAR target cells were completely eliminated only at the highest *E*:*T* ratio tested. Thus, the prerequisite for an efficient T-cell-based αCAR therapy is that the *E*:*T* ratio is in favor of the αE-tag effector T cells.

### Effector cells upregulate CAR expression upon target T-cell killing

**Fig. 4:**
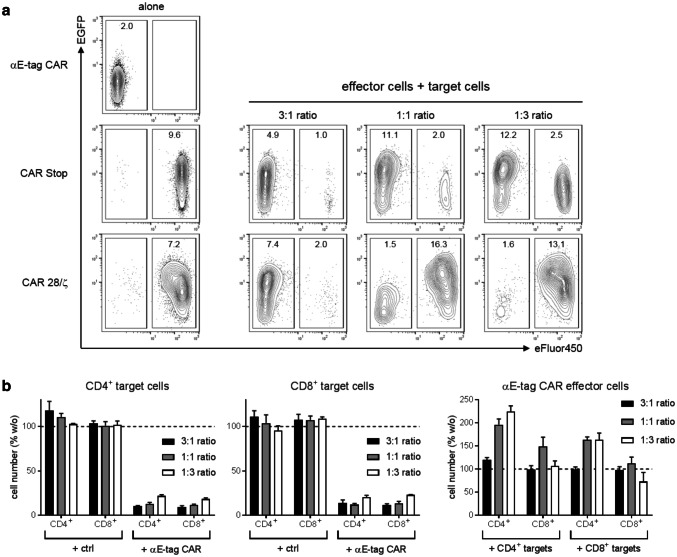
Analysis of CAR expression levels and killing efficiencies of T-cell subpopulations. **a** Numbers in contour plots represent MFI of EGFP for both αE-tag CAR effector cells (eFluor™450^−^) and CAR Stop or CAR 28/ζ target cells (eFluor™450^+^) after 2 days of coculture. Results are reported for one representative donor out of three. **b** CD4^+^ and CD8^+^ CAR Stop T cells were incubated at indicated *E*:*T* ratios with αE-tag CAR effector or CAR Stop (ctrl) T cells. After 48 h, CD4^+^ and CD8^+^ target and effector cells were quantified. Absolute numbers of T cells cultured alone were set to 100% and corresponding relative cell number in the presence of effector/target cells was calculated. Error bars represent SD of triplicate wells. Results for one representative donor are shown

In previous studies, we observed that the expression of co-translated EGFP marker protein directly correlates with surface expression of CAR constructs [e.g., 23, 24]. Usually, we verify CAR T-cell expression via an αE-tag mAb (supplementary Fig. 3). As αE-tag CARs lack any extracellular tags, we monitored EGFP signal of T cells as a surrogate for CAR expression level changes (Fig. 4a). Upon activation, effector cells strongly upregulated the expression of their αE-tag CAR as shown for cocultivation with CAR Stop-modified target cells at all tested *E*:*T* ratios as well as for a 3:1 ratio with CAR 28/ζ-armed T cells. Vice versa, target cells that receive an activating signal via their intracellular 28/ζ domain upon cross-linkage to effector cells also increased CAR expression (Fig. 4a, lower panel, 1:1 and 1:3 ratio). When analyzing the remaining E-tag-labeled T cells it becomes obvious that they showed reduced EGFP levels compared to conditions in which they were cultured in the absence of effector cells [e.g., CAR Stop: 9.6 vs. 1.0, 2.0 and 2.5 median fluorescence intensity (MFI) of EGFP at 3:1, 1:1 and 1:3 ratio, respectively]. This might be caused by the downregulation of the E-tag-containing CAR, yet, a more likely explanation is the preferential killing of T cells displaying a high CAR expression than of cells expressing only low levels of E-tag-comprising CARs. To substantiate this hypothesis, we analyzed elimination kinetics of target cells that were presorted into CAR low- and CAR high-expressing subpopulations based on the strength of their EGFP signal (supplementary Fig. 4). Compared to their EGFP^low^ counterpart, target T cells exhibiting high CAR densities were indeed more efficiently killed. In accordance, expansion of αE-tag CAR effector T cells that were incubated with EGFP^high^ target T cells was more pronounced than in cocultures with sorted EGFP^low^ cells. Together, these data support our assumption that high E-tagged CAR expression results in preferential target cell killing.

### CD4^+^ and CD8^+^ target T cells are equally well depleted by CD4^+^ and CD8^+^ effector T cells

As we established a functional αCAR CAR for efficient depletion of genetically modified T cells, we wanted to use this construct as a tool for investigating CD4^+^ and CD8^+^ T-cell killing capabilities in more detail. For that purpose, we isolated and transduced both subpopulations separately with either an E7B6-tagged CAR Stop or an αE-tag CAR. In subsequent flow cytometry-based cytotoxicity assays over 48 h, both CD4^+^ and CD8^+^ effector cells performed equally well in killing of E-tag-containing CAR target T cells (Fig. 4b). However, the CD4^+^ T-cell subset demonstrated a stronger proliferative capacity compared to CD8^+^ T cells, most likely due to the fact that the latter produce less growth-promoting IL-2 than T-helper cells. Again, monitoring the EGFP signal indicates that effector cells vigorously enhanced the expression of their αE-tag CAR upon activation (supplementary Fig. 5a) and preferentially eliminated target cells with the high expression of the E-tag containing CAR (supplementary Fig. 5b). The cytotoxicity data further reveal that CD4^+^ and CD8^+^ cells can be targeted efficiently with no apparent differences between the two T-cell populations (Fig. 4b).

To shed some light on the mode of action, intracellular staining for granzymes were conducted (supplementary Fig. 6a). As CD8^+^ T cells were already highly positive for GzmA in a resting state, upon cocultivation with target cells only GzmB was strongly upregulated. In contrast, CD4^+^ T-cell activation induced expression of both GzmA and GzmB. Interestingly, we could detect elevated granzyme levels in target cells (supplementary Fig. 6b). As they were not expressing CD107a (see also Fig. 3c), we reason that GzmA and GzmB are not produced by target cells themselves but originated from effector cells and were transported via the cytolytic immune synapse. Further evidence to support this notion was obtained by staining target T cells that were cocultured with mock-transduced T cells instead of cytolytic effector cells. As shown in supplementary Fig. 6c, enhanced GzmA/GzmB was only detectable in E-tagged CAR T cells that were targeted by αE-tag CAR effector T cells, whereas granzyme levels of target T cells cocultured with mock control resembled baseline expression.

### Killing of CAR T cells results in the abrogation of their effector functions in vitro and in vivo

**Fig. 5:**
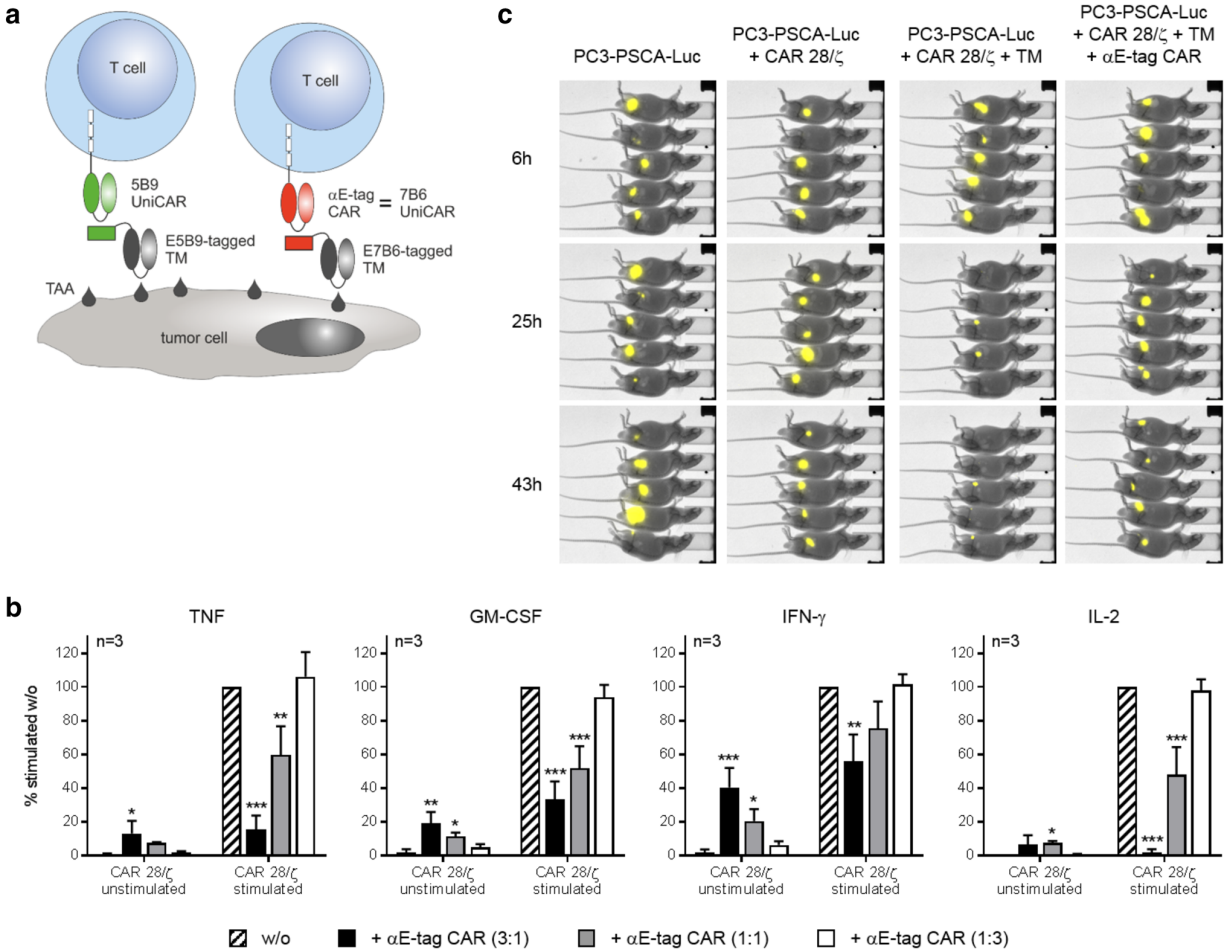
Impairment of effector functions of E-tagged CAR 28/ζ T cells. **a** The physiologically inactive 5B9 UniCAR is switched on by a tumor-associated antigen (TAA)-binding TM comprising the peptide epitope E5B9 (left). Similarly, via E7B6-tagged TMs, αE-tag CAR T cells can be repurposed as alternative 7B6 UniCAR T cells (right). **b** CAR 28/ζ target T cells were left unstimulated or redirected to PC3-PSCA tumor cells using an αPSCA-E5B9 TM (stimulated). For target T-cell killing under both conditions, αE-tag CAR effector T cells were added at indicated *E*:*T* ratios for 72 h. Cytokine levels in the presence of αE-tag CAR T cells were normalized to samples with stimulated CAR 28/ζ T cells alone. (*p < 0.05, **p < 0.01, ***p < 0.001 with respect to CAR 28/ζ target T cells w/o effector T cells, one-way ANOVA with Bonferroni multiple comparison test). **c** Bioluminescence imaging of anesthetized mice was conducted at indicated time points post tumor cell injection. Control animals were transplanted with PC3-PSCA-Luc tumor cells alone or together with CAR 28/ζ T cells. Treatment groups additionally received an αPSCA-E5B9 TM in the presence or absence of autologous αE-tag CAR T cells (*E*:*T* ratio 3:1)

Having successfully shown that αCAR-engineered T cells attack E-tagged CAR T cells, we next wanted to clarify how this influences effector functions of the targeted lymphocytes. As readout, secretion of proinflammatory cytokines in vitro as well as tumor cell killing in vivo was chosen. As already stated above, one kind of E-tagged CARs are our switchable and flexible modular UniCARs [24, 26, 27, 32, 35–38]. Utilizing the La/SS-B-derived peptide epitope E5B9 as linker, the platform technology enables a precisely controlled activation of otherwise inert 5B9 UniCAR-armed T cells by infusion of small E5B9-tagged TMs (Fig. 5a, left panel). For proof-of-concept, we used these UniCAR cells as targets for αCAR-equipped cells and stimulated them against the tumor-associated antigen PSCA via an αPSCA-E5B9 TM. Activation resulted in the substantial release of proinflammatory TNF, GM-CSF and IFN-γ as well as mitogenic IL-2 (Fig. 5b, striped bar). Addition of αE-tag CAR T cells resulted in a dose-dependent reduction of secreted cytokines. Interestingly, retargeting of αCAR T cells against the E-tag did not trigger profound cytokine production (Fig. 5b and supplementary Fig. 7) which, in light of a putative clinical translation, is beneficial since it can be reasoned that T-cell-mediated CAR T-cell killing does not exacerbate cytokine release syndrome. As depicted in Fig. 5c, E-tagged UniCAR 28/ζ T cells were not only eliminated in vitro but also in vivo. NMRI^nu/nu^ mice were transplanted with Luc-expressing PC3-PSCA cells and survival of tumor cells was monitored by means of bioluminescence imaging. Two days after the injection of Luc^+^ PC3-PSCA cells, UniCAR 28/ζ T cells and a cross-linking αPSCA-E5B9 TM, Luc activity was almost completely cleared. By contrast, anti-tumor activity was markedly impaired upon co-injection of αE-tag CAR T cells (Fig. 5c, right panel) verifying that αCAR-modified T cells are functional in experimental mice post-transfer and efficiently kill E-tagged target T cells in vivo.

### αE-tag CAR-engineered T cells can be repurposed for antitumor treatment in vitro and in vivo

**Fig. 6:**
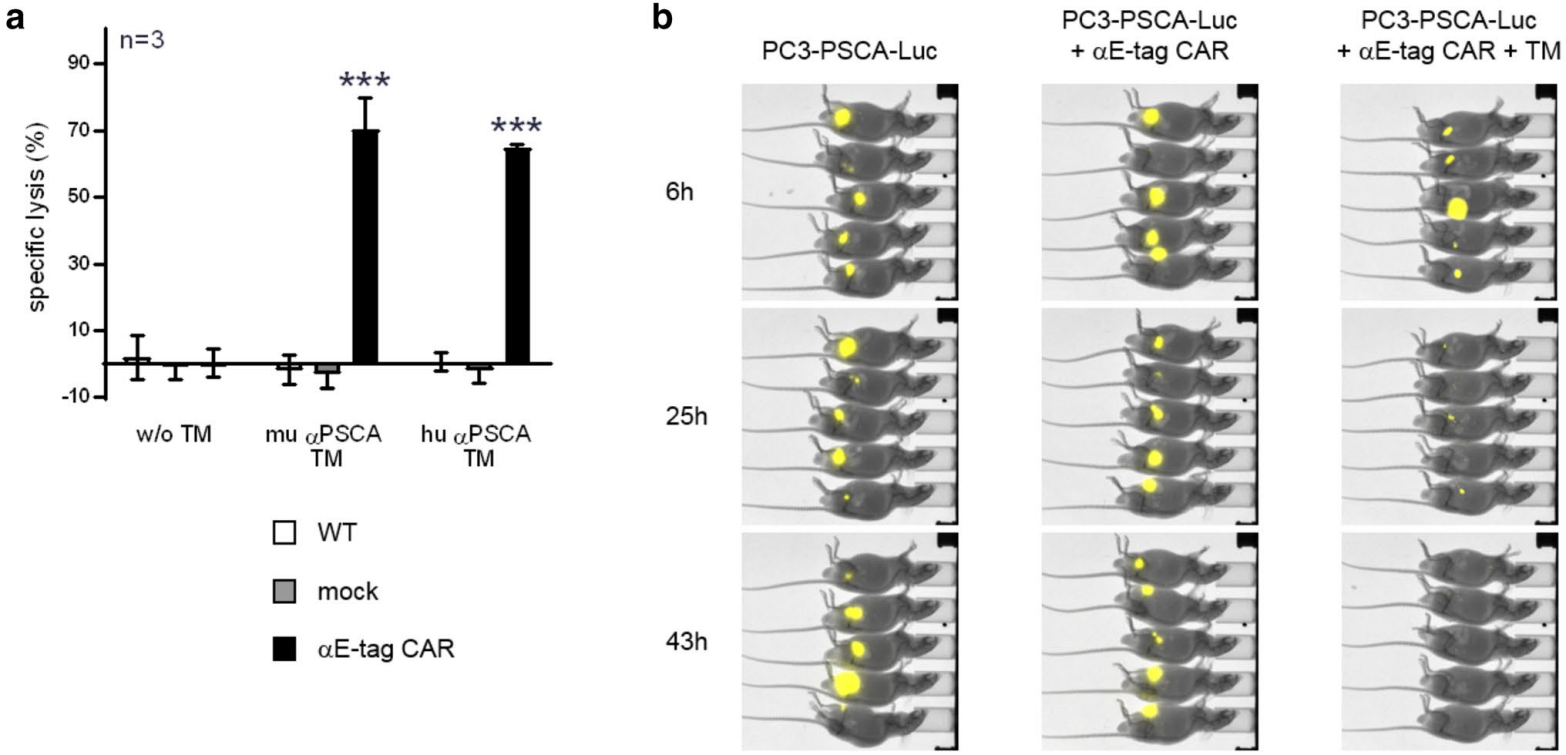
Killing of tumor cells by αE-tag CAR T cells redirected via E7B6-tagged TMs. **a** αE-tag CAR T cells were incubated with chromium-labeled PC3-PSCA tumor cells (*E*:*T* ratio of 5:1) either with or without a murine (mu) or humanized (hu) αPSCA-E7B6 TM. Non-transduced T cells (WT) or T cells expressing only EGFP (mock) served as negative control. After 48 h, tumor cell lysis was assessed (***p < 0.001, two-way ANOVA with Bonferroni multiple comparison test). **b** Bioluminescence images of mice that were s.c. injected with a mixture of Luc-expressing PC3-PSCA tumor cells and αE-tag CAR T cells with or without a hu αPSCA-E7B6 TM. Tumor-only control animals are the same as presented in Fig. 5c, due to experiments being performed in parallel

The complete eradication of a therapeutic CAR T-cell population inevitably entails termination of antitumor activity. In contrast to other currently available safety strategies, the αCAR CAR methodology provides the unique possibility to re-initiate tumor treatment as the αE-tag CAR T cells can be repurposed as tumor-fighting CAR T cells. As schematically summarized in Fig. 5a, they may even replace the original therapeutic cell product and function as an alternative UniCAR variant which we termed ‘7B6 UniCAR’ T cells. Like the original UniCARs which are directed against the peptide epitope E5B9, 7B6 UniCARs can be redirected by administering E-tag-comprising TMs (Fig. 5a, right panel). To support this idea, we generated two novel TMs consisting of either a murine or a humanized scFv against PSCA fused to the E-tag. Indeed, as demonstrated in Fig. 6a, these TMs efficiently mediated cross-linkage of αE-tag CAR T cells and PSCA-expressing PC3 cells, resulting in significant tumor cell lysis in vitro. The TM-dependent anti-tumor response of αE-tag CAR-modified T cells could furthermore be substantiated in vivo (Fig. 6b). To that end, athymic nude mice were injected with Luc-expressing PSCA^+^ tumor cells either alone or together with αE-tag CAR T cells with or without the humanized PSCA-binding E-tagged TM. After 2 days, Luc activity was no longer detectable in the treatment group (Fig. 6b, right panel) whilst tumor cells were still visible in both control groups (Fig. 6b, left and middle panel). Taken together, αE-tag CAR T cells can not only trigger elimination of autologous CAR T cells but can function as an alternative UniCAR variant if required.

## Discussion

Although gene-modified CAR T cells have demonstrated unparalleled antitumor responses in hematological malignancies [2–6], adoptive therapy is often associated with severe, partly life-threatening side effects [4–11]. These side effects can be divided into short-time and long-time effects. For example, soon after the start of a CAR T-cell therapy, the adoptively transferred T cells can rapidly expand potentially leading to a severe cytokine storm. In addition, the simultaneous disintegration of a huge number of tumor cells may cause a tumor lysis syndrome. After removal of the tumor cells still side effects may occur due to cross-reactivity of the anti-tumor domain of the CAR with healthy tissues as even seen with the CD19 CAR. For these reasons, safeguards to control toxicity are required. There are currently two major ideas followed to control such severe side effects: (i) elimination of CAR T cells and (ii) engineering of tunable CAR T cells based on molecular switches and/or combinatorial targeting according to the rules of Boolean algebra [41, 42]. The activity of switchable CARs can be controlled, e.g., by soluble tumor-specific Ab-based components such as the TMs in the UniCAR system [24, 26, 27, 32, 35–38] or even chemically tuned via small molecules (ON-switch CARs) [43]. Bearing the high costs of CAR T-cell products in mind, approaches aiming to fine-tune CAR T cells should be favored in the first place. However, it would be advantageous to have an additional safeguard available if switchable CARs do not behave as predicted. Although unlikely for terminally differentiated cells and not observed until now, one should always keep in mind that genetic manipulation of cells harbors the risk to generate leukemic CAR T cells either by accidentally transducing single leukemic cells during the manufacturing process or by insertional mutagenesis. In case such leukemic CAR T cells develop, elimination strategies for both conventional and switchable CAR T cells are highly required.

Here, we report an efficient chance to selectively deplete genetically engineered T cells by autologous αCAR-redirected T cells. For this purpose, we have integrated a specific peptide epitope (E7B6) into the CAR architecture that can be used as an inherent elimination tag. Based on a mAb recognizing this tag, an αE-tag CAR construct was designed and successfully generated. Using flow cytometry-based cytotoxicity assays we demonstrate that T cells equipped with this novel CAR selectively bind and eliminate CAR T cells with an incorporated E-tag whilst CAR T cells lacking this tag are not attacked.

Even though the CD8^+^ subpopulation has long been considered as the most potent T-cell subtype in terms of cytotoxicity, our killing data reveal that CD4^+^ effector T cells eliminate target cells equally efficient via granzyme-mediated apoptosis. One likely explanation is that our read-out is performed after 24 h and 48 h, whilst other studies perform short-term cytotoxicity assays over 4 h [e.g., 44]. In that regard, we have previously published that the onset of killing via CD4^+^ T cells is delayed, with no obvious effects seen 5 h or 6 h after stimulation. Yet, upon prolonging incubation to 20 h, a substantial cytotoxic activity comparable to that of CD8^+^ T cells is detectable [34, 45].

Our data further indicate that target cells expressing high CAR levels are more prone to depletion by αE-tag redirected effectors than cells with low CAR expression. This is in accordance with previously published studies reporting a positive correlation between the number of EGFR cell-surface molecules and cetuximab-triggered ADCC [18, 46]. However, in the aforementioned study and other currently investigated safety strategies, the suicide/elimination marker is separated from the CAR construct [13–18, 20]. Given that T cells with low safeguard molecule expression display a reduced susceptibility to apoptosis, CAR-engineered T cells lacking the suicide/elimination marker might emerge due to selective pressure. These cells ultimately escape control and perpetuate toxicity. By contrast, integrating the E-tag into the extracellular spacer region of the CAR should prevent CAR T-cell escape. An additional benefit of this approach is the small size of the peptide epitope of only 18 aa. Opposed to that, other proposed depletion markers are relatively large proteins that add a substantial payload to the expression vector potentially interfering with transcriptional efficiency. Most recently, a study reported the development of a so-called CubiCAR, combining three functions (detection, purification and depletion) in one CAR molecule by incorporating CD20 mimotopes and a CD34 epitope into the extracellular region of a CAR [47]. Although not investigated in the present manuscript, also the E-tag has utility beyond function as elimination marker. As previously shown, it enables detection, selective ex vivo expansion and purification of E-tagged CAR T cells prior to adoptive transfer [23]. In that regard, we reason that in vivo tracking of gene-modified cells using for example radio-labeled mAbs against the E-tag is feasible as well.

Despite the fact that all currently available elimination markers, in contrast to suicide genes, possess multifunctional characteristics [17–20, 47], several drawbacks are associated with the use of mAbs. First, the efficacy of CAR T-cell depletion via ADCC might be impaired in heavily preconditioned cancer patients due to their compromised immune system. Second, cetuximab and rituximab inevitably provoke on-target adverse effects upon recognition of healthy epidermal tissue and endogenous B cells, respectively. In this regard, using an epitope derived from the human nuclear La/SS-B protein provides a safe approach as the targeted antigen is not available on the surface of intact cells under physiological conditions [28, 48]. Third, to ensure therapeutic efficiency, a sufficiently high local Ab concentration is required which might not be achieved in all tissues due to limited penetration and retention of infused mAbs. In light of this and given that T cells elicit highly potent cytotoxic effector mechanisms, αCAR-redirected T cells represent a promising approach to bypass the above-discussed shortcomings of drug-based depletion strategies. In contrast to mAbs, T cells engraft and proliferate, hence, can be considered as self-amplifying “living drugs”. Furthermore, they show better tissue penetration and might, therefore, be superior to mAbs in eliminating CAR T cells. Considering the high costs of CAR T-cell products, clinical application of αCAR T cells as a safety switch may be particularly relevant (i) if other mechanisms/strategies fail to control serious treatment-related toxicities or (ii) if leukemic CAR T cells occur.

Nevertheless, once tumor-specific CAR T cells are completely wiped out, therapeutic antitumor activity cannot be retrieved. However, our proposed safety approach offers the unique feature of repurposing αCAR-engineered T cells as tumor-fighting UniCAR T cells by administration of an E-tag-comprising TM. Thereby, tumor treatment can be re-initiated on demand in case of relapse, which represents a substantial advantage over all currently available safeguard strategies.

In summary, we provide first experimental evidence for using αCAR-redirected T cells for selective elimination of gene-modified T cells in case of life-threatening CAR therapy-related side effects. For specific recognition of CAR-expressing T cells, we integrated a small epitope tag (E-tag) into the CAR architecture without impairing functionality of the original construct. This E-tag then serves as a targetable moiety for specific and stringent CAR T-cell depletion via autologous αCAR-engrafted T cells. As the E-tag can be incorporated into all CARs irrespective of the targeted tumor antigen, it represents a promising universal tool to enhance safety of all kinds of cell-based immunotherapies.

### Electronic supplementary material

Below is the link to the electronic supplementary material.

Supplementary material 1 (PDF 885 kb)

## Abbreviations

ADCC: Antibody-dependent cellular cytotoxicity
CDC: Complement-dependent cytotoxicity
CHO: Chinese hamster ovary
EGFP: Enhanced green fluorescent protein
E-tag: Peptide epitope E7B6
GzmA: Granzyme A
GzmB: Granzyme B
HEK293T: Human embryonic kidney cell line
Luc: Firefly luciferase
MFI: Median fluorescence intensity
Orf: Open reading frame
PSCA: Prostate stem cell antigen
TM: Targeting module
UniCAR: Universal chimeric antigen receptor
*V*_H_: Variable domain of the heavy chain of a monoclonal antibody
*V*_L_: Variable domain of the light chain of a monoclonal antibody
