# Supplementary material for: Anti-CAR-engineered T cells for epitope-based elimination of autologous CAR T cells
Source: Cancer Immunol Immunother. 2019 Aug 14;68(9):1401–15. doi: 10.1007/s00262-019-02376-y (PMC6768917; doi:10.1007/s00262-019-02376-y)
Supplement: Supplementary file 1 — Supplementary material 1 (PDF 885 kb) [file 262_2019_2376_MOESM1_ESM.pdf]

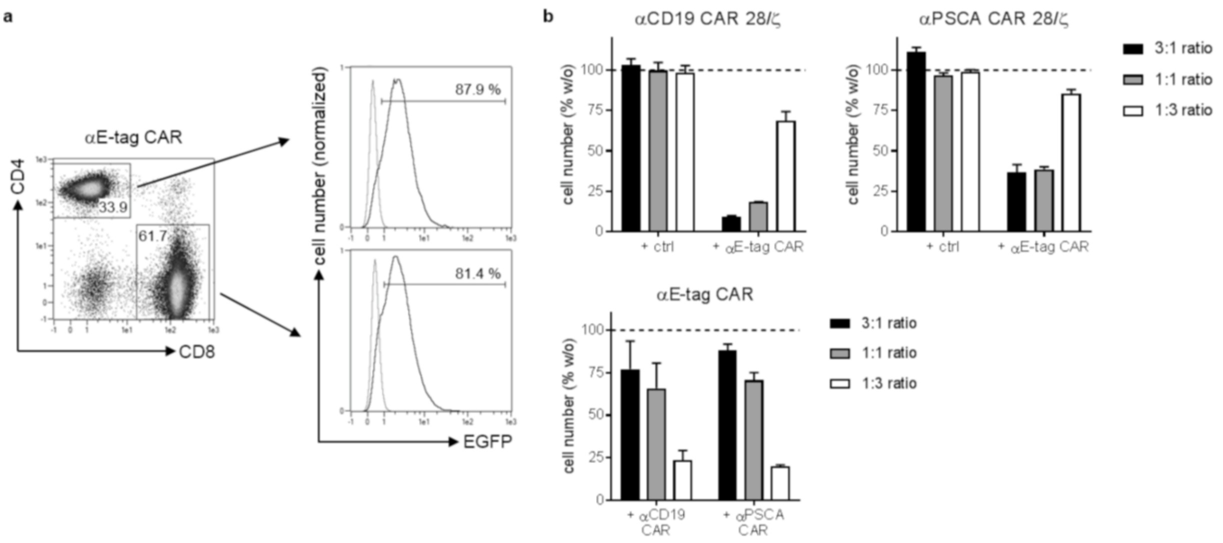

Supplementary Figure 1 αE-tag CAR T cells induce killing of E-tagged CAR target cells. **a** Transduction efficiency of αE-tag CAR T cells was assessed by analysis of co-translated EGFP marker protein. Histograms show percentage of $EGFP^+CD4^+$ (upper panel) and $EGFP^+CD8^+$ (lower panel) T cells. Non-transduced T cells (grey graph) served as negative control. Staining example of one out of four different T cell donors is shown. **b** E-tagged CD19- or PSCA-specific CAR T cells were cultured with αE-tag CAR effector or mock-transduced T cells (+ ctrl) at indicated ratios. Cell number of αCD19 (n=2, upper left panel) or αPSCA (n=1, upper right panel) target T cells as well as αE-tag CAR effector T cells (lower panel) was measured after 24h. Absolute T cell numbers cultured alone were set to 100% and relative cell number in the presence of effector/target cells was calculated

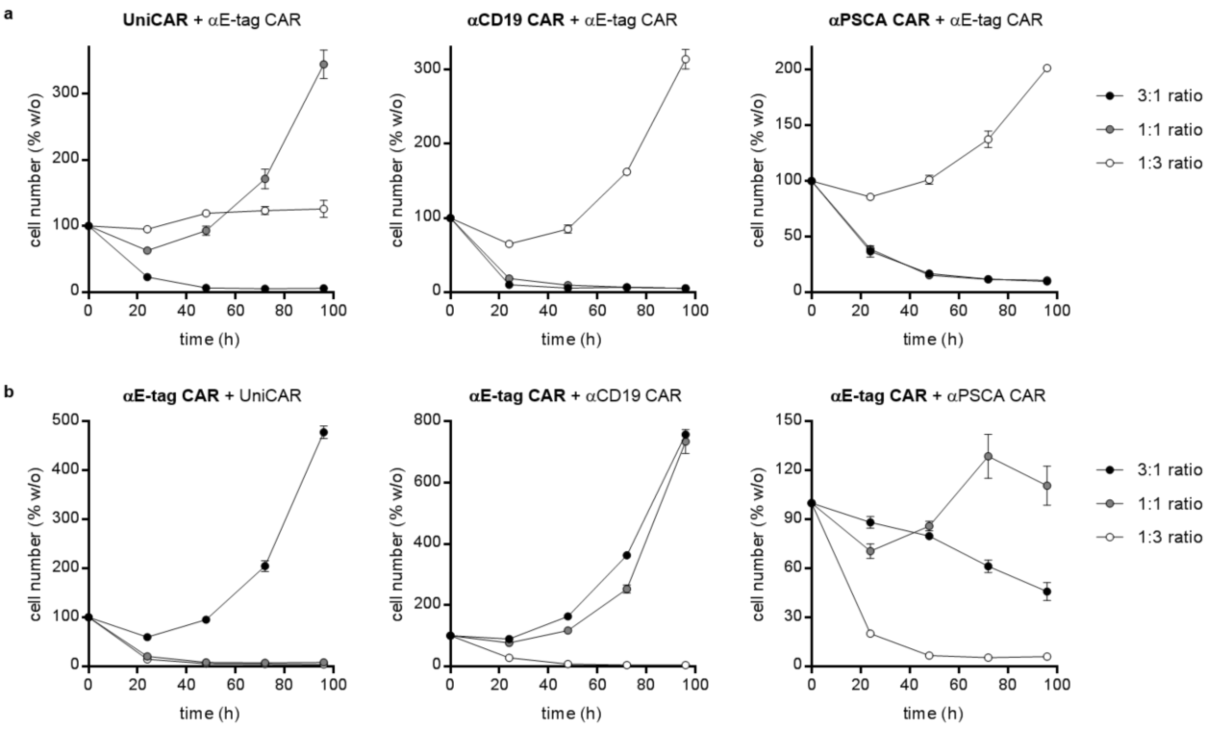

Supplementary Figure 2 Long-term monitoring of target and effector CAR T cell numbers in cocultures. In a flow cytometry-based cytotoxicity assay, αE-tag CAR effector T cells were incubated with T cells expressing different E-tagged CAR constructs (UniCAR, αCD19 CAR, αPSCA CAR) at indicated E:T ratios. Absolute numbers of T cells cultured alone were equalized to 100% and corresponding percentage of **a** target cells or **b** effector cells in cocultures was calculated. Results for triplicates of one out of two representative donors are shown

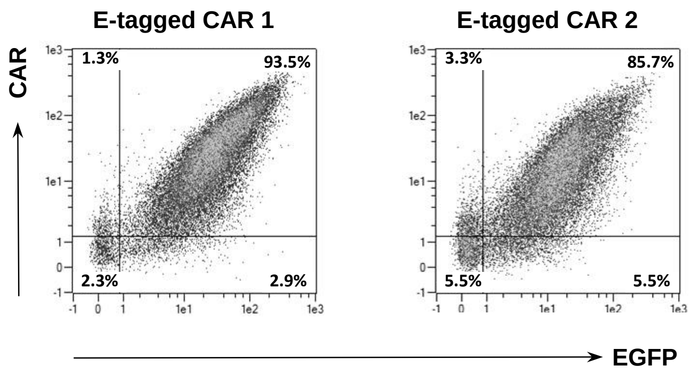

Supplementary Figure 3 Correlation of EGFP and CAR expression of genetically modified T cells. Cells were stained with an αE-tag mAb and a secondary goat anti-mouse IgG F(ab')2-PE Ab to verify surface expression of the E-tagged CAR. CAR surface expression is plotted against co-translated EGFP marker protein. Data of one representative donor are shown

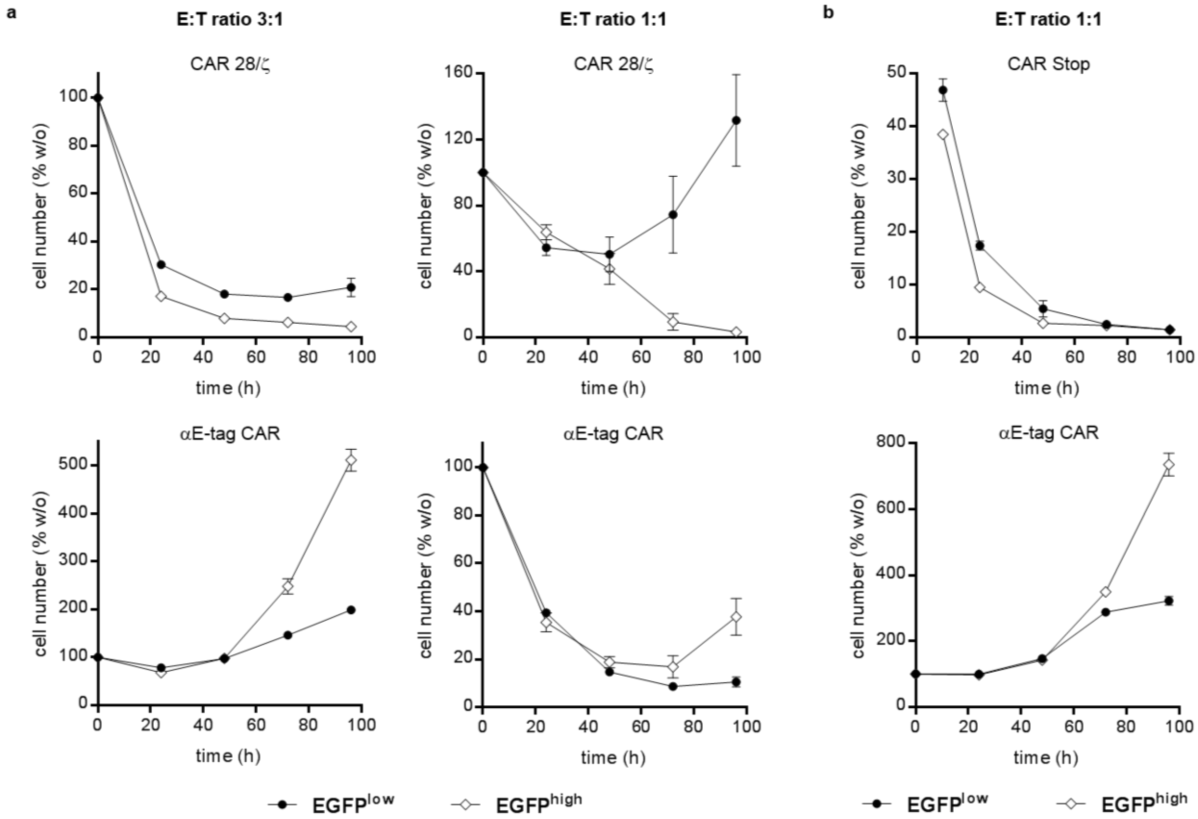

Supplementary Figure 4 Improved killing of E-tagged target T cells expressing high CAR levels. One week after transduction, E-tagged **a** CAR 28/ζ T cells or **b** CAR Stop T cells lacking the intracellular signaling domain were sorted for $EGFP^{low}$ or $EGFP^{high}$ expressing cells. Over 4 days, target (upper row) and αE-tag CAR effector (lower row) T cells were cocultured and repeatedly quantified by flow cytometry. Absolute number of T cells cultured alone was set to 100% and corresponding percentage of cells in coculture with effector/target cells was calculated. Results for triplicates of one representative donor are depicted

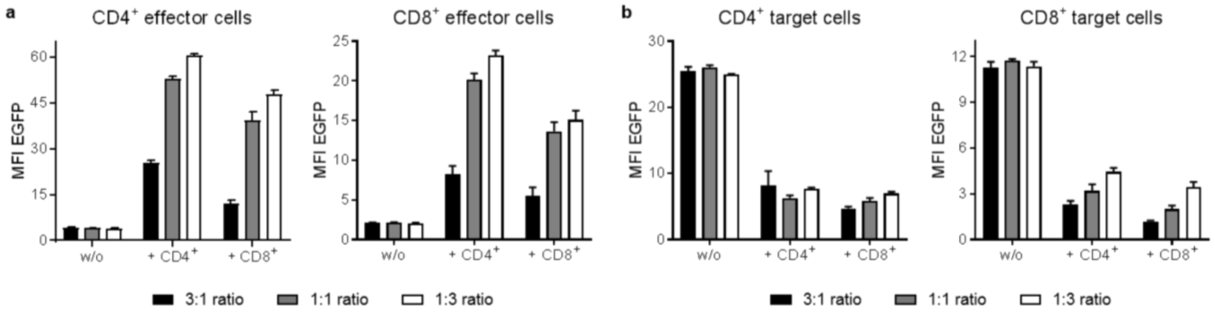

Supplementary Figure 5 Changes in CAR expression levels of CD4$^+$ and CD8$^+$ effector and target T cells. Both CD4$^+$ and CD8$^+$ T cells were engrafted with an αE-tag CAR (effector cells) or an E-tag-containing CAR Stop (target cells). After two days of coculture at indicated ratios, median EGFP signal as surrogate for CAR expression of **a** EGFP$^+$eFluor™450$^-$ effector and **b** EGFP$^+$eFluor™450$^+$ target cells was assessed by flow cytometry. Results are shown for triplicates of one representative donor

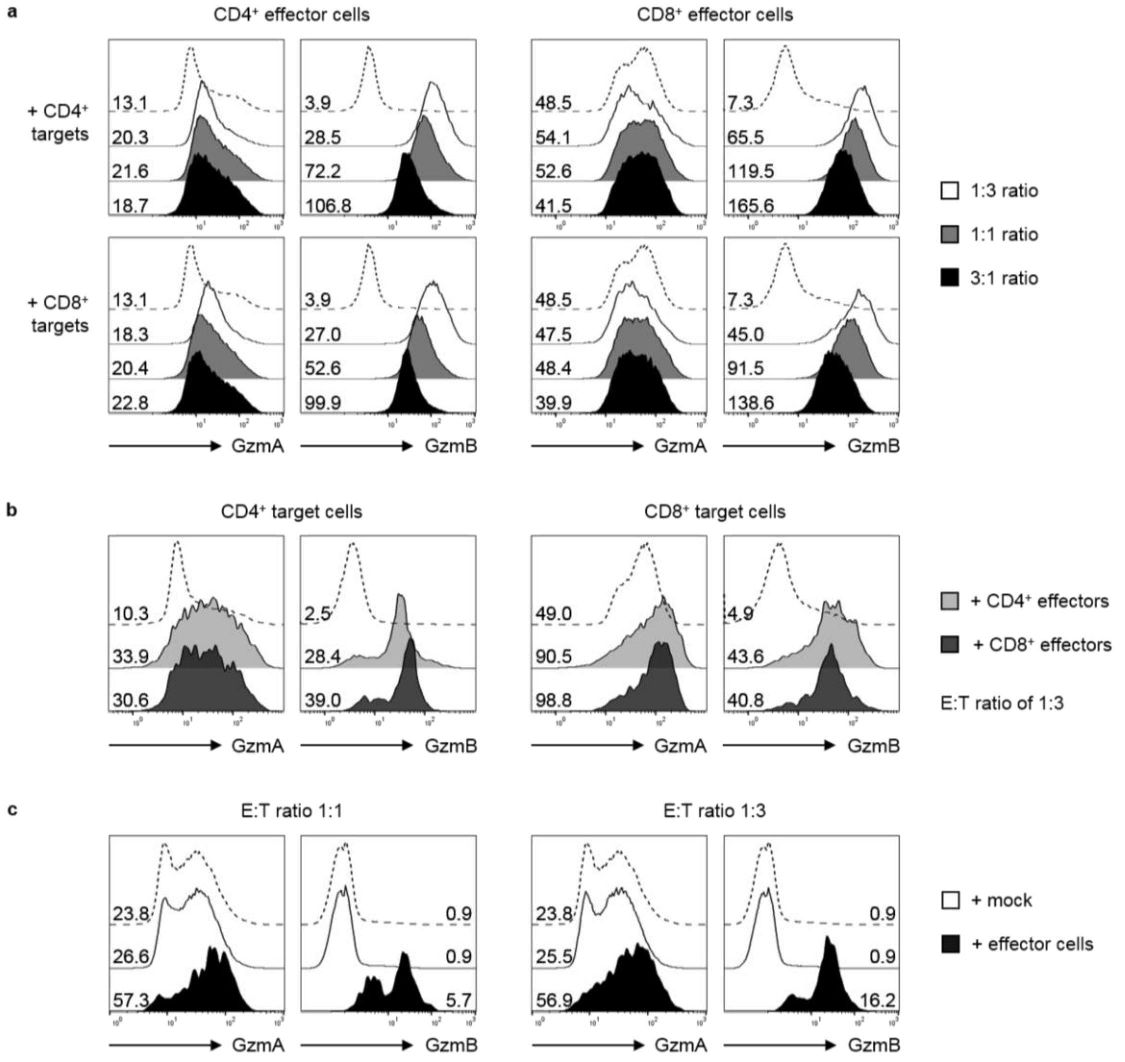

Supplementary Figure 6 Upregulation of granzyme expression by αE-tag CAR-activated T cells. Both CD4+ and CD8+ T cell subpopulations were genetically modified to express an αE-tag CAR (effector cells) or an E-tagged CAR Stop (target cells). After 48h of coculture at indicated ratios, intracellular GzmA and GzmB expression of **a** EGFP+eFluor™450− effector and **b** EGFP+eFluor™450+ target cells was measured by flow cytometry. **c** E-tag comprising CAR Stop T cells were incubated with αE-tag CAR effector or mock-transduced control T cells. Intracellular GzmA and GzmB levels of eFluor™450-labeled target cells were detected two days later. **a-c** T cells cultured alone (dashed line) were used to assess baseline granzyme expression levels. Numbers represent MFI of total cells for each condition. Results for one representative donor are depicted

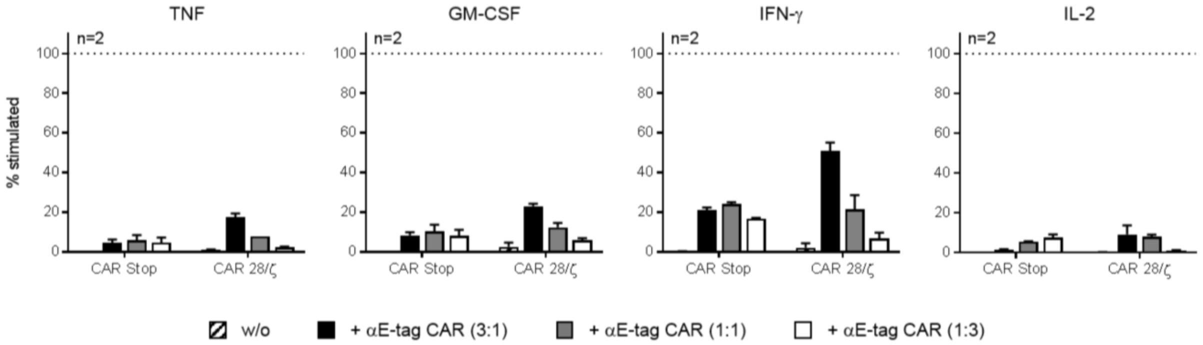

Supplementary Figure 7 Cytokine secretion by E-tag-redirected αCAR effector T cells. For stimulation of αE-tag CAR cells, cocultures at indicated E:T ratios with E-tagged CAR target cells either lacking (CAR Stop) or comprising (CAR 28/ζ) an intracellular signaling domain were performed. Concentration of TNF, GM-CSF, IFN-γ, and IL 2 secreted within 72h of coculture was assessed by ELISA. Detected cytokine levels were normalized to cytokine production of tumor cell-redirected, autologous CAR 28/ζ target T cells. Summarized data of two independent donors are depicted as mean ± SD
